# Salivary Chemical Barrier Proteins in Oral Squamous Cell Carcinoma—Alterations in the Defense Mechanism of the Oral Cavity

**DOI:** 10.3390/ijms241713657

**Published:** 2023-09-04

**Authors:** Gergő Kalló, Petra Magdolna Bertalan, Ildikó Márton, Csongor Kiss, Éva Csősz

**Affiliations:** 1 Proteomics Core Facility, Department of Biochemistry and Molecular Biology, Faculty of Medicine, University of Debrecen, Egyetem tér 1, 4032 Debrecen, Hungary; bertalan.petra@med.unideb.hu (P.M.B.); marton.ildiko@dental.unideb.hu (I.M.); cseva@med.unideb.hu (É.C.); 2 Biomarker Research Group, Department of Biochemistry and Molecular Biology, Faculty of Medicine, University of Debrecen, Egyetem tér 1, 4032 Debrecen, Hungary; 3 Doctoral School of Molecular Cell and Immune Biology, University of Debrecen, Egyetem tér 1, 4032 Debrecen, Hungary; 4 Division of Pediatric Hematology-Oncology, Department of Pediatrics, Faculty of Medicine, University of Debrecen, Nagyerdei krt. 98, 4032 Debrecen, Hungary; kisscs@med.unideb.hu

**Keywords:** oral squamous cell carcinoma, OSCC, antimicrobial and immunomodulatory proteins, AMP, saliva, chemical barrier

## Abstract

Oral squamous cell carcinoma (OSCC) is one of the most frequent types of head and neck cancer. Despite the genetic and environmental risk factors, OSCC is also associated with microbial infections and/or dysbiosis. The secreted saliva serves as the chemical barrier of the oral cavity and, since OSCC can alter the protein composition of saliva, our aim was to analyze the effect of OSCC on the salivary chemical barrier proteins. Publicly available datasets regarding the analysis of salivary proteins from patients with OSCC and controls were collected and examined in order to identify differentially expressed chemical barrier proteins. Network analysis and gene ontology (GO) classification of the differentially expressed chemical barrier proteins were performed as well. One hundred and twenty-seven proteins showing different expression pattern between the OSCC and control groups were found. Protein–protein interaction networks of up- and down-regulated proteins were constructed and analyzed. The main hub proteins (IL-6, IL-1B, IL-8, TNF, APOA1, APOA2, APOB, APOC3, APOE, and HP) were identified and the enriched GO terms were examined. Our study highlighted the importance of the chemical barrier of saliva in the development of OSCC.

## 1. Introduction

The oral cavity is one of the most frequent sites of head and neck cancers, developing predominantly as oral squamous cell carcinoma (OSCC) in the upper aerodigestive epithelium [1,2]. OSCC accounts for nearly 95% of all cancer types in the oral cavity, 2% of all malignant lesions, and more than 30,000 new cases per year worldwide [3]. OSCC mainly affects the elderly population since the average age of diagnosis is around 60 years with an approximately 2:1 male:female ratio [4]. In Europe, the age-standardized rates for both the incidence and mortality of oral cavity and pharyngeal cancers are high, without substantial improvements in the last decades [5]. Among the European countries, Hungary has the highest rate of incidence and mortality of OSCC [5]. OSCC is frequently being diagnosed in advanced stages and, despite considerable progress in surgical methods, radio-, chemo-, and immunotherapy, the long-term survival rate is around 50%. In contrast, the recovery rates for patients with early stage lesions may be up to 80% [6]. Unfortunately, with the exception of biopsy, considered as the gold standard procedure, there are no evidence-based, reliable, non-invasive methods for large-scale screening and early detection of OSCC [7]. Besides genetic risk factors, such as the overexpression of *NPM*, *CDK1,* and *NDRG1* genes and underexpression of *CHES1* [8], tobacco and alcohol consumption and poor oral hygiene are also risk factors in the carcinogenesis of OSCC [9,10,11]. Microbial infections and/or dysbiosis are also associated with the development of OSCC, and the impact of HPV infection was highlighted as well [12]. Studies have shown that bacteria, such as *Porphyromonas gingivalis, Fusobacterium nucleatum, Peptostreptococcus, Filifactor, Parvimonas, Pseudomonas, Campylobacter*, and *Staphylococcus* species, can participate in the development of OSCC [13]. Moreover, Robayo et al. highlighted the importance of the co-infection of HPV and *Streptococcus anginosus* in the development of OSCC [14].

Saliva is a complex mixture of organic and inorganic compounds continuously secreted from major and minor salivary glands and the gingival crevice [15]. It is a very dilute body fluid composed of approximately 99% water with varying (0.7–2.4 µg/µL) protein concentrations [16], showing high variability depending on the age, sex, sample collection time, and health status of the oral cavity. Saliva contains more than 2700 proteins, and the most abundant proteins belong to the antimicrobial and immunomodulatory protein (AMP) family [17]. AMPs are elements of the innate immune system [18] and constitute the first line of defense in protecting the host from invading pathogens by creating a chemical barrier [19]. In the human body, the chemical barriers contain several prototypic AMPs, such as defensins, dermcidin, and LL-37 cathelicidin [20], and there are several proteins with much higher concentrations compared with prototypic AMPs. These proteins, e.g., lactotransferrin, lipocalins, lysozyme-C, lacritin, etc., belong to the highly abundant body fluid proteins, with various defense functions [20]. The non-invasive collection and continuous availability of saliva make it an excellent target for omics and biomarker studies. Unsurprisingly, the protein composition of saliva has been analyzed by several workgroups aiming to identify new biomarkers, indicating its relevance to medical applications [21]. Since OSCC is one of the most frequent tumor types in the oral cavity and the analysis of salivary proteins has a high impact, many studies have been carried out in order to identify the alterations in the saliva of patients with OSCC compared with controls [22,23,24,25]. Our workgroup also demonstrated changes in the proteome and transcriptome of saliva from patients with OSCC [26,27,28,29].

Considering that saliva contains many chemical barrier proteins and the fact that OSCC can alter the protein composition secreted into saliva, our aim was to analyze the effect of OSCC on the salivary chemical barrier proteins. We collected publicly available datasets regarding the analysis of salivary proteins from patients with OSCC and controls and we searched for differentially expressed chemical barrier proteins. Network and GO analyses of the differentially expressed chemical barrier proteins were performed as well.

## 2. Results

In our study, we reutilized publicly available datasets in order to analyze salivary proteins from patients with OSCC and matched controls originating from the ProteomeXchange [30] and PubMed [31] repositories. We searched for chemical barrier proteins in the downloaded datasets in the UDAMP database previously created by our workgroup [32] and we searched for chemical barrier proteins with significantly different expression between OSCC and control groups. Our evaluation relied on the results of the statistical analyses performed by the authors.

### 2.1. Differential Expression of Chemical Barrier Proteins in Saliva of Patients with OSCC

Our analysis revealed that 94 chemical barrier proteins showed significantly elevated amounts in the saliva of patients with OSCC compared with controls. From the 94 proteins that showed elevated expression, 77 AMPs (Table 1), 10 complement system proteins (Table 2), and 7 cytokines (Table 3) were identified.

Besides the upregulated salivary chemical barrier proteins, we identified 28 AMPs with significantly lower amounts in the saliva of patients with OSCC compared with the controls (Table 4). All downregulated proteins belonged to the AMP family.

Considering the number of chemical barrier proteins that showed altered expression between patients with OSCC and controls, the presence of the tumor in the oral cavity could alter the expression profile and/or secretion of AMPs and other barrier components, and could also alter the defense function of the barrier.

From the set of differentially expressed chemical barrier proteins identified by our meta-analysis, we found five salivary proteins where the data were contradictory. In some cases, the authors reported significantly elevated amounts in patients with OSCC, while other studies suggested decreased expression of the proteins compared with controls (Table 5).

We examined the clinical data presented by the authors of the studies, and one of the possible reasons for the contradictory data could be the different grades and stages of the tumors. The clinical data suggest that the localization of the tumor in the oral cavity has a slight effect on the secreted chemical barrier proteins. Moreover, the difference between the studied cohorts was also a possible reason for the contradictory data, since the secreted components of saliva can vary between different populations.

### 2.2. Network Analysis of Chemical Barrier Proteins Affected by OSCC

In order to gain more insight into the biological consequences of the changes in the chemical barrier composition, the up- and down-regulated chemical barrier proteins were further subjected to network analysis. We used the STRING-DB (version 11.5) [117] and Cytoscape software (version 3.9.1) [118] to map the interactions between the proteins. Furthermore, to gain more insights into the biological functions of the revealed AMPs, we performed gene ontology (GO) analyses using ClueGO (version 2.5.9) [119] Cytoscape plug-in.

**Figure 1:**
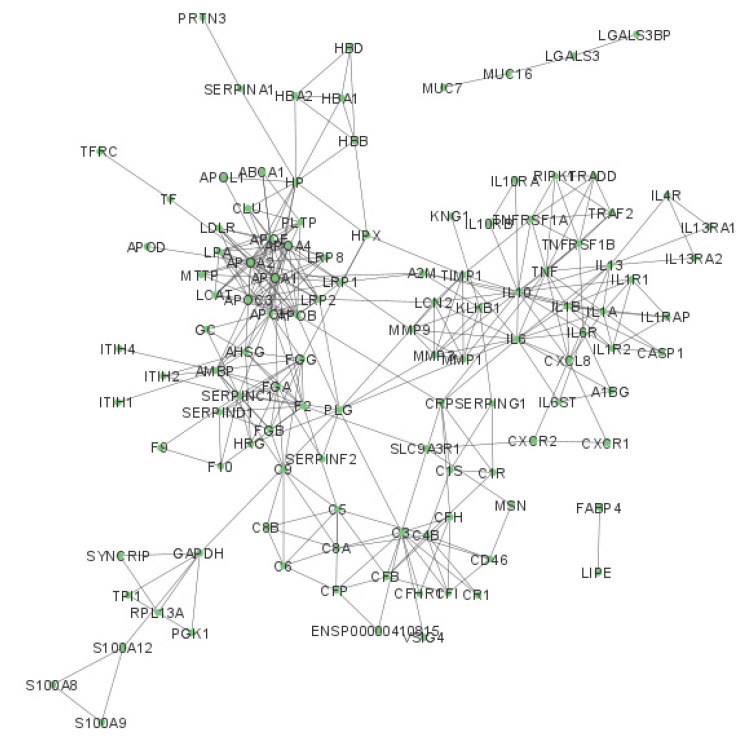
The interaction network of the upregulated chemical barrier proteins in saliva from patients with OSCC. Each circle represents a protein and the lines indicate interactions. The proteins are labeled with their gene name. The high-resolution image of this network is presented in Figure S1.

The protein–protein interaction networks of the up-regulated salivary chemical barrier proteins in OSCC with their 50 first shell interactors are presented in Figure 1 and Figure S1.

The analysis of the chemical barrier proteins with their first shell interactors detected 116 proteins with 334 connections. The network analysis revealed that most of the chemical barrier proteins were part of two core clusters that interacted with each other. Many different proteins with high numbers of interactions are present, such as apolipoprotein A1, antithrombin III, IL-6, and complement C3. On the other hand, we identified two additional clusters with a small number of proteins (FABP4 and LIPE; MUC7, MUC16, LGALS3, and LGALS3BP) and without interactions with the core network.

**Figure 2:**
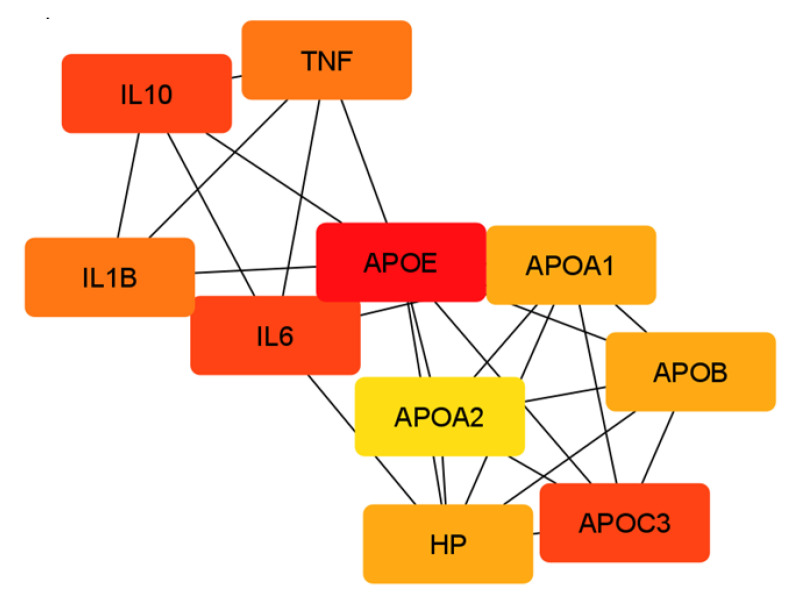
Top 10 hub proteins in the network of salivary chemical barrier proteins upregulated in OSCC. The proteins are labeled with their gene name. A darker color means a higher number of connections.

In order to identify the top hub proteins in the network of the chemical barrier proteins altered by OSCC, the datasets were analyzed by CluePedia [120] and CytoHubba [121]. The top 10 identified hub proteins in the network of up-regulated proteins in OSCC are represented in Figure 2.

The hub proteins observed in the network of the up-regulated proteins were mainly apolipoproteins, cytokines, and haptoglobin (Figure 2), indicating their important function in the chemical barrier of the oral cavity.

**Figure 3:**
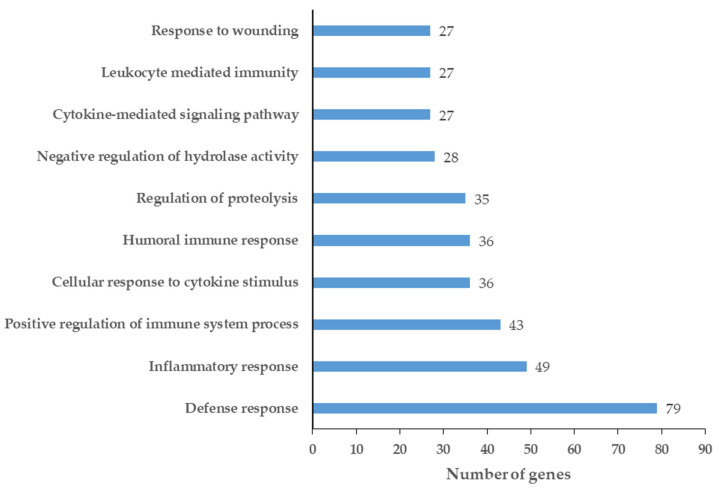
Top 10 enriched GO terms for chemical barrier proteins up-regulated in the saliva of patients with OSCC. The enriched GO terms were ordered according to the gene count.

To obtain functional information, the enriched GO terms were examined using ClueGO (Table S1), and the top 10 enriched GO terms were visualized in Figure 3.

The GO functions enriched in the network of up-regulated proteins (Figure 3) were mainly related to defense mechanisms, such as defense, inflammatory, and humoral immune responses. However, the cellular immune response, cytokine signaling pathways, wounding, and the regulation of protease activity were observed as well.

The data support that, during tumor development, the humoral immune responses are activated, creating an inflammatory environment that has already been linked to the development of OSCC [122,123,124].

**Figure 4:**
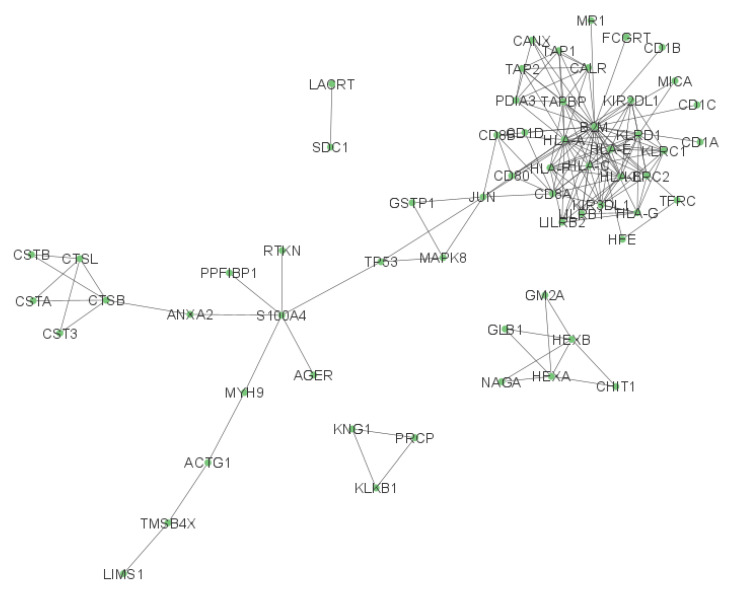
Network view of the interaction network of the down-regulated chemical barrier proteins in saliva from patients with OSCC. Each circle represents a protein, and the lines indicate interactions. The proteins are labeled with their gene name. The high-resolution image of this network is presented in Figure S2.

We also examined the network of the chemical barrier proteins down-regulated in the saliva of patients with OSCC (Figure 4 and Figure S2).

The network analysis identified 61 proteins with 158 connections and revealed one complex cluster that included most of the chemical barrier proteins in connection with most of the remaining AMPs. We also identified three additional small clusters (LACRT and SDC1; KNG1, KLKB1, and PRCP; GM2A, GLB1, NAGA, HEXA, HEXB, and CHIT1) without interactions with the core network. Compared with the network obtained from the up-regulated proteins, the network of the down-regulated chemical barrier proteins in OSCC showed a lower number of protein clusters.

The analysis of the top 10 hub proteins was also performed on the down-regulated proteins, but only the first shell interactor proteins were present as hubs.

**Figure 5:**
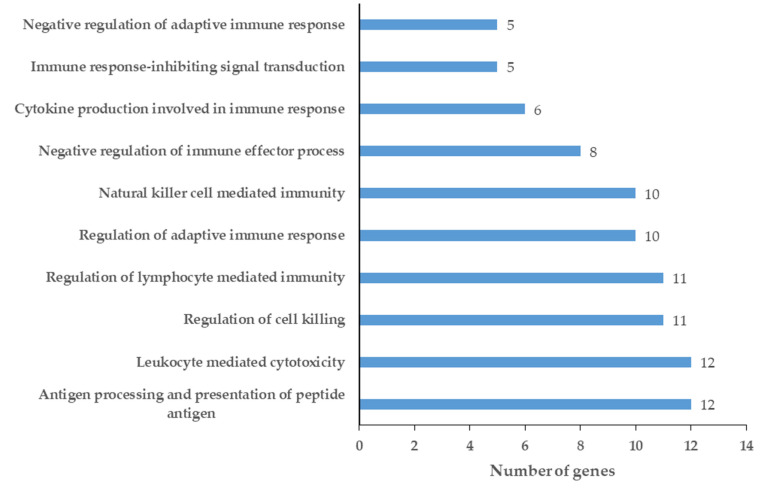
Top 10 enriched GO terms for chemical barrier proteins down-regulated in the saliva of patients with OSCC. The enriched GO terms were ordered according to the gene count.

The enriched GO terms in the network of down-regulated proteins were also examined (Table S2), and the top 10 enriched GO terms were visualized, as shown in Figure 5.

In the case of the network of down-regulated proteins, the enriched GO terms were mainly related to the adaptive and cellular immune responses, suggesting alterations in the immune response during tumor development.

## 3. Discussion

OSCC is a common type of head and neck carcinoma with high incidence and prevalence, creating a socio-economical burden. The mortality rate of the disease is high, mainly due to late diagnosis. Since the survival rate of the disease is low, it is extremely important to better understand the molecular mechanisms behind the progression and development of OSCC [125]. In this study, we aimed to examine the chemical barrier proteins in the saliva of patients with OSCC and controls in order to investigate the effect of the tumor on the barrier of the oral cavity. Therefore, the scientific literature was reviewed and searched for datasets regarding the analysis of the protein content of saliva from the above-mentioned groups.

Altogether, we collected 30 datasets from the PubMed and ProteomeXchange repository that examined the differences in the salivary proteins of patients with OSCC and healthy controls. We collected the differentially expressed proteins based on the statistical analyses applied by the authors, and we identified the components of the salivary chemical barrier by searching in the UDAMP database. After evaluation, we found 127 proteins that showed different expression patterns between the OSCC and control groups. Of the 127 proteins, 94 were up-regulated (Table 1, Table 2 and Table 3) and 28 proteins were down-regulated in OSCC compared with controls (Table 4). We also found five proteins with contradictory expression profiles in the two groups; several studies indicated up-regulation and other studies indicated down-regulation of these five proteins (Table 5). Most of the up-regulated proteins belonged to the AMP family, but cytokines and complement system proteins were identified as well. In the case of the down-regulated proteins, only the members of the AMP family were identified.

### 3.1. Amylases and Mucins in the Chemical Barrier of Patients with OSCC

Amylases and mucins are the most abundant proteins in the saliva [17], maintaining the homeostatic functions, but also parts of the chemical barrier. Owing to their hydrolytic activity, amylases can alter the biofilm formation of bacteria by cleaving the polysaccharide backbone of extracellular polymeric structures [97]. However, evidence shows that amylase can bind to the amylase-binding protein of *Streptococcus* species and can induce biofilm formation [97]; therefore, the effect of amylases in the formation of bacterial biofilms is still not clear. Mucins are high-molecular-weight glycoproteins also acting in the chemical barrier. MUC5B and MUC7 can interact with a variety of bacteria, such as *Streptococcus* species and *Pseudomonas aeruginosa*, and pathogenic fungi, such as *Candida albicans*, to prevent the activity and further invasion of these microorganisms [77]. Our analyses revealed elevated levels of MUC7 and MUC16 in the saliva of patients with OSCC compared with controls (Table 1), indicating their role in tumor progression. We also identified that the level of amylases was down-regulated in patients with OSCC compared with controls (Table 4). Since the major salivary proteins are affected by OSCC, further study would be necessary to gain more insight into the role of these proteins in cancer development.

### 3.2. Proteases and Protease Inhibitors in the Salivary Chemical Barrier of Patients with OSCC

Besides amylases, other hydrolases, such as proteases, are also affected by OSCC. Proteases and protease inhibitors are constitutive parts of each chemical barrier of the human body. A variety of cells express and secrete a wide range of proteolytic enzymes in order to defend the host against potential pathogens by the degradation of proteins involved in the life cycle of pathogens. Saliva contains a broad spectrum of proteases and, since salivary proteases were recognized as potential biomarkers for oral cancer [126], they have high relevance in the homeostatic and pathological processes of the oral cavity. Since proteases are double-edged swords capable of degrading host proteins as well, protease inhibitors are crucial for the host. While the protease inhibitors of the host provide a defense against their own proteases, they can also inhibit the proteases secreted by pathogenic microorganisms [127], indicating their important role in chemical barriers. The list of the differentially expressed chemical barrier proteins in OSCC contained many proteases. The levels of MMP1, MMP9, myeloblastin, prostasin, and stromelysin-1 were elevated in the saliva of patients with OSCC, while lower amounts of ER aminopeptidase 2, kallikrein 11, and lysosomal Pro-X carboxypeptidase were identified compared with controls (Table 1 and Table 4). Many protease inhibitors, such as SERPINs and inter-alpha-trypsin inhibitor heavy chains, showed elevated expression profiles in OSCC (Table 1), while other protease inhibitors, like cystatins and SPINK5, showed reduced expression profiles in OSCC compared with controls (Table 4). Our results indicate that the proteolytic and anti-proteolytic activity of the saliva is altered in patients with OSCC; however, the alteration may not shift the balance between the proteases and protease inhibitors, since the effect of up-regulated proteins can be balanced with the down-regulation of other proteases and protease inhibitors.

### 3.3. Contribution of Cytokines in the Salivary Chemical Barrier of Patients with OSCC

The intention to maintain the balance in homeostatic functions can also be observed in the case of up-regulated pro- and anti-inflammatory cytokines. Cytokines are small proteins secreted by a variety of cells and have specific effects on the interactions and communications between cells. Cytokines can fulfill autocrine, paracrine, or endocrine actions and have pleiotropic effects on target cells [96]. The fact that cytokines are part of the top 10 hub proteins in the network of chemical barrier proteins up-regulated in OSCC (Figure 2) highlights the importance of these proteins in tumor development. Pro-inflammatory cytokines, such as IL-1α, IL-1β, IL-6, and TNF, showed higher amounts in the saliva of patients with OSCC, indicating the involvement of the inflammatory environment in tumor progression [128]. Additionally, our workgroup demonstrated that IL-6 is a robust biomarker for OSCC in saliva [28]. However, along with the pro-inflammatory cytokines, the levels of Il-10 and IL-13 anti-inflammatory cytokines were upregulated in OSCC as well (Table 3). This suggests that the body tries to fight against the inflammatory pathways activated by the pro-inflammatory cytokines and tries to keep the balance between the pro- and anti-inflammatory processes.

### 3.4. Members of S100 Protein Family Are Affected by OSCC

Besides cytokines, other proteins, such as S100 proteins, can also participate in the regulation of cellular responses. Members of the S100 family are Ca^2+^-binding proteins with potent antimicrobial activity against pathogens [129]. The secreted forms of S100 proteins also have paracrine effects by regulating different cell types, such as immune cells, endothelial cells, and muscle cells [129]. Our analysis revealed that eight S100 proteins (S100A2, S100A7, S100A7A, S100A8, S100A9, S100A11, S100A12, and S100P) were up-regulated in the saliva of patients with OSCC compared with controls (Table 1), while a lower amount of S100A4 was identified in OSCC compared with controls (Table 4). S100A7, or psoriasin, is a well-known potent AMP that mainly can be found on the surface of the skin [20]. S100A9 was also identified by our workgroup as a potential salivary biomarker of OSCC [26]. Our examination revealed that many S100 proteins are affected by OSCC, indicating their important role in the pathomechanism of this type of cancer.

### 3.5. OSCC Can Enhance the Complement System in the Salivary Chemical Barrier

While the different mediators can activate a variety of cellular responses, the humoral immune response can be activated as well, such as antibody production by plasma cells or the activation of the complement system. The complement system is part of the immune system that enhances the clearance of microorganisms and damaged cells, promotes inflammatory responses, and disrupts the cell membrane of pathogens [95]. The complement system is composed of several small proteins mainly synthesized by the liver in precursor forms [95]. The activation of the complement proteins follows a cascade model; after activation, proteases in the system cleave their targets and start the cascade system. Our analysis revealed 10 complement system proteins that were up-regulated in the saliva of patients with OSCC compared with controls (Table 2). Complement proteins are already associated with oral cancer [130], and evidence suggests that they have important roles in the tumor microenvironment as well [131]. Therefore, complement proteins are possible targets for anticancer therapies [131]. Our network analysis highlighted that some complement proteins, such as complement C3 and complement C9, are hub proteins that are important in communication with the other subnetworks (Figure 1).

### 3.6. Elevated Levels of Apolipoproteins in the Saliva of Patients with OSCC

The levels of many apolipoprotein forms, such as Apo AI, Apo B-100, Apo D, and Apo E, were elevated in the saliva of patients with OSCC (Table 1). The main function of these proteins is the construction of lipoproteins, such as chylomicron, VLDL, LDL, or HDL, that carry triglycerides, cholesterol, cholesterol esters, and other types of lipids in the circulation system [132]. Besides their important role in lipid transport, apoproteins also take part in host defense mechanisms. The antimicrobial activity of Apo A1 against *Staphylococcus epidermidis* has been described [42] and antimicrobial peptides derived from the further processing of Apo B acting against *Salmonella* strains have also been identified [133]. Our results indicate that apolipoproteins are important hub proteins in the protein–protein interaction network (Figure 2), highlighting the importance of apoproteins in the chemical barrier of the oral cavity.

### 3.7. Salivary Chemical Barrier Proteins with Contradictory Expression Profile in OSCC

Among proteins that were clearly up- or down-regulated in the saliva of patients with OSCC, five AMPs were found to be differentially expressed between OSCC and control groups, but the way that they changed was contradictory (Table 5). There is evidence in the scientific literature of either the up- and down-regulation of these salivary proteins in OSCC. The possible reason for this contradiction may be the different stages of the tumors and the difference between the studied cohorts. One of our previous studies highlighted the importance of population-tailored studies [26]. Depending on the different sex, age, and ethnicity of the studied cohorts, the expression profiles of the salivary chemical barrier proteins may be different from each other.

### 3.8. Alterations in the Defense Mechanism of the Oral Cavity

Oral microorganisms, including bacteria, archaea, fungi, viruses, and protozoa, are closely associated with the physiological/pathological state of the oral cavity. Currently, more than 1000 bacterial species are known in the oral cavity [134], including *Actinobacteria*, *Bacteroidetes*, *Chlamydia*, *Euryarchaeota*, *Fusobacteria*, *Firmicutes*, *Proteobacteria*, *Spirochaetes*, and *Tenericutes* species [135]. Along with the classical proteomics studies [136], metaproteomics approaches have emerged for examining the connections between the proteome of the oral cavity and the proteome of the oral microbiota [137,138,139]. Studies described that oral microbiome dysbiosis can lead to the development of pathological changes in the oral cavity, such as caries and periodontal diseases [140], and is also associated with systemic diseases, such as obesity, diabetes [134], lung cancer [137], and oral cancer [134]. Our analysis revealed that OSCC can alter the secretion of many chemical barrier proteins in the saliva and, as the tumor alters the protein content of the chemical barrier, the defense mechanism of the oral cavity can change, allowing uncontrolled proliferation of distinct microbial species. Data in the literature highlight that HPV, *Porphyromonas gingivalis, Fusobacterium nucleatum, Peptostreptococcus, Filifactor, Parvimonas, Pseudomonas, Campylobacter,* and *Staphylococcus* species are known pathogens that participate in the development of OSCC [13,14]. One of the possible reasons for the dysbiosis could be the change in the defense function of the chemical barrier caused by the altered expression of the defense and regulatory proteins. As OSCC is still an emerging problem in our society, it is extremely important to better understand the biological events leading to tumor progression. The analysis of the chemical barrier proteins and their interactions with the oral microbiome can highlight additional layers of the host–tumor interactions and can be important for the design of new possible therapies. Therefore, further studies are needed to investigate the defense mechanism of the saliva against the above-mentioned pathogens involved in tumor development.

### 3.9. Limitations of the Study

In this study, we revealed that the differential expression and/or secretion of chemical barrier proteins into saliva could be linked to OSCC and we highlighted the importance of this protein family in the progression of OSCC. However, the majority of the data related to differentially expressed proteins originated from survey studies, and most of the changes have not been validated yet. Therefore, the validation of the changes in these proteins will be an important task in further studies. While the available databases contain a huge amount of information, our knowledge of protein–protein interactions is constantly improving, revealing more and more interaction partners of specific proteins. Thus, our interaction networks represent our current knowledge of the interactions between the proteins making up the chemical barrier and may change when databases are further updated. Our study focused only on the composition of the salivary chemical barriers; however, analyses of cellular responses [141] and small molecules secreted into saliva [142] can add additional layers to our knowledge of the development and progression of OSCC.

## 4. Materials and Methods

### 4.1. Examination of Chemical Barrier Proteins in OSCC Datasets

Datasets involving human saliva samples from patients with OSCC and healthy controls were retrieved from PubMed [31] and ProteomeXchange [30] until May 2023. Datasets were selected for examination if samples from patients with OSCC and matched controls were examined and their comparison was carried out. Altogether, 30 datasets were selected for examination (Table 6).

The chemical barrier proteins were searched in the downloaded datasets by using the UDAMP database [32]. Those chemical barrier proteins whose level showed a statistically significant change in OSCC samples compared with controls were listed and assigned for further analyses. Our evaluation relied on the results of the statistical analysis performed by the authors of the datasets.

### 4.2. Network Analysis

In order to investigate the biological processes relevant to the differentially expressed chemical barrier proteins, network analyses were performed using the STRING-DB (v11.5) [117] and Cytoscape (v3.9.1) [118] software, along with the ClueGO (v2.5.9) [119], CluePedia (v1.5.9) [120], and CytoHubba (v0.1) [121] plug-ins.

In order to create and analyze the interaction networks of the differentially expressed chemical barrier proteins, the STRING-DB and Cytoscape software programs were used, as described by Kumar et al. [32]. The differentially expressed proteins originated from the statistical analyses performed by the authors. Briefly, interaction networks of the differentially expressed proteins and up to 50 of their first shell interactors were retrieved. The top 10 genes based on their network degree were imported into ClueGO in order to examine the GO terms enriched in the networks of chemical barrier proteins. The gene names were uploaded and, after species selection (Homo sapiens [9606]), “Functional Analysis” in the “Analysis Mode” menu was used and pathways were searched by setting the “Significance” in the “Visual Style” menu and to “Show only Pathways with pV ≤ 0.05000”. All the other settings remained as default. The top 10 enriched GO terms were selected based on the number of detected genes. The gene visualization threshold for the CluePedia analysis was set to 1000 and the other analysis parameters were set to default. The identification of hub proteins was performed using the CytoHubba plug-in and the top 10 nodes ranked by degree were selected.

## 5. Conclusions

In this study, we highlighted the importance of the chemical barrier of saliva in the development of OSCC. As pathogenic microorganisms can participate in the development of OSCC, the alteration in the defense function of the saliva may contribute to tumor progression. Our study can serve as a starting point for further examinations regarding the possible link between the altered defense mechanism and tumor progression.

## Figures and Tables

**Table 1:** AMPs with increased amount in saliva of patients with OSCC compared with controls. The functions of proteins in the chemical barrier are indicated as well.

Uniprot Entry	Protein Name	Function in the Chemical Barrier	Reference
P02763	Alpha-1-acid glycoprotein 1	Immunomodulatory effect	[33]
P01011	Alpha-1-antichymotrypsin	Protease inhibitor	[34]
P01009	Alpha-1-antitrypsin	Protease inhibitor	[35]
P04217	Alpha-1B-glycoprotein	Immunomodulatory effect	[36]
P08697	Alpha-2-antiplasmin	Protease inhibitor	[37]
P02765	Alpha-2-HS-glycoprotein	Immunomodulatory effect	[38]
P01023	Alpha-2-macroglobulin	Protease inhibitor	[39]
P03973	Antileukoproteinase	Protease inhibitor/Immunomodulatory effect	[40]
P01008	Antithrombin-III	Protease inhibitor	[41]
P02647	Apolipoprotein A-I	Antimicrobial activity	[42]
P02652	Apolipoprotein A-II	Immunomodulatory effect	[42]
P06727	Apolipoprotein A-IV	Immunomodulatory effect	[43]
P04114	Apolipoprotein B-100	Antimicrobial activity	[44]
P02656	Apolipoprotein C-III	Immunomodulatory effect	[45]
P05090	Apolipoprotein D	Immunomodulatory effect	[46]
P02649	Apolipoprotein E	Immunomodulatory effect	[47]
O14791	Apolipoprotein L1	Immunomodulatory effect	[48]
P02749	Beta-2-glycoprotein 1	Immunomodulatory effect	[49]
Q9NP55	BPI fold-containing family A member 1	Antimicrobial activity	[50]
P04040	Catalase	Antibacterial activity	[51]
P00450	Ceruloplasmin	Antimicrobial/Cu sequestration	[52]
P10909	Clusterin	Immunomodulatory effect	[53]
P08185	Corticosteroid-binding globulin	Protease inhibitor	[54]
P02741	C-reactive protein	Antimicrobial activity/Acute-phase protein	[55]
P12724	Eosinophil cationic protein	Antimicrobial activity	[56]
P15090	Fatty acid-binding protein 4	Immunomodulatory effect	[57]
P02671	Fibrinogen alpha chain	Antimicrobial activity	[58]
P02675	Fibrinogen beta chain	Antimicrobial activity	[58]
P02679	Fibrinogen gamma chain	Antimicrobial activity	[58]
Q08380	Galectin-3-binding protein	Immunomodulatory effect/Antimicrobial activity	[59,60]
P06396	Gelsolin	Processed from has antimicrobial activity	[61]
P78417	Glutathione S-transferase omega-1	Immunomodulatory effect	[62]
P04406	Glyceraldehyde-3-phosphate dehydrogenase	Immunomodulatory effect	[63]
P00738	Haptoglobin	Immunomodulatory effect/iron sequestering	[64]
P00739	Haptoglobin-related protein	Antiparasitic activity	[65]
P69905	Hemoglobin subunit alpha	Processed forms (hemocidins) have antimicrobial activity	[66]
P68871	Hemoglobin subunit beta	Processed forms (hemocidins) have antimicrobial activity	[66]
P02042	Hemoglobin subunit delta	Processed forms (hemocidins) have antimicrobial activity	[66]
P02790	Hemopexin	Immunomodulatory effect/ Antimicrobial activity	[67]
P05546	Heparin cofactor 2	Protease inhibitor	[68]
P04196	Histidine-rich glycoprotein	Antimicrobial activity	[69]
Q96QV6	Histone H2A type 1-A	Antimicrobial activity	[70]
P19827	Inter-alpha-trypsin inhibitor heavy chain H1	Protease inhibitor	[71]
P19823	Inter-alpha-trypsin inhibitor heavy chain H2	Protease inhibitor	[71]
Q14624	Inter-alpha-trypsin inhibitor heavy chain H4	Protease inhibitor	[71]
P01042	Kininogen-1	Antimicrobial activity	[72,73]
P03956	Matrix metalloproteinase-1	Protease activity	[74]
P14780	Matrix metalloproteinase-9	Protease activity/Protective role against bacterial infections	[75]
P26038	Moesin	Immunomodulatory effect	[76]
Q8WXI7	Mucin-16	Antimicrobial activity	[77]
Q8TAX7	Mucin-7	Antimicrobial activity	[77]
P24158	Myeloblastin	Protease activity	[78]
P80188	Neutrophil gelatinase-associated lipocalin	Immunomodulatory effect/iron sequestration	[79]
O75594	Peptidoglycan recognition protein 1	Antimicrobial activity	[80]
P36955	Pigment epithelium-derived factor	Protease inhibitor	[81]
P05155	Plasma protease C1 inhibitor	Protease inhibitor	[82]
P13796	Plastin-2	Immunomodulatory effect	[83]
Q16651	Prostasin	Protease activity	[84]
P02760	Protein AMBP	Protease inhibitor	[85]
P31949	Protein S100-A11	Immunomodulatory effect	[86]
P80511	Protein S100-A12	Immunomodulatory effect	[87]
P29034	Protein S100-A2	Immunomodulatory effect	[88]
P31151	Protein S100-A7	Immunomodulatory effect	[88]
Q86SG5	Protein S100-A7A	Immunomodulatory effect	[88]
P05109	Protein S100-A8	Immunomodulatory effect	[88]
P06702	Protein S100-A9	Immunomodulatory effect	[88]
P25815	Protein S100-P	Immunomodulatory effect	[88]
O95969	Secretoglobin family 1D member 2	Immunomodulatory effect	[88]
P02787	Serotransferrin	Antimicrobial/Iron sequestration	[89]
P48594	Serpin B4	Protease inhibitor	[82]
E9PGN7	Serpin family G member 1	Protease inhibitor	[82]
P35542	Serum amyloid A-4 protein	Immunomodulatory effect	[90]
P02743	Serum amyloid P-component	Antiviral activity	[90]
P08254	Stromelysin-1	Protease activity	[91]
P05543	Thyroxine-binding globulin	Protease inhibitor	[92]
P37802	Transgelin-2	Immunomodulatory effect	[93]
P02774	Vitamin D-binding protein	Immunomodulatory effect	[94]

**Table 2:** Complement system components with increased amounts in saliva of patients with OSCC compared with controls. The functions of the proteins in the chemical barrier are indicated as well.

Uniprot Entry	Protein Name	Function in the Chemical Barrier	Reference
P00736	Complement C1r subcomponent	Opsonization of bacteria/Immunomodulatory effect	[95]
B4E1Z4	Complement C2	Opsonization of bacteria/Immunomodulatory effect	[95]
P01024	Complement C3	Opsonization of bacteria/Immunomodulatory effect	[95]
P0C0L5	Complement C4-B	Opsonization of bacteria/Immunomodulatory effect	[95]
P01031	Complement C5	Opsonization of bacteria/Immunomodulatory effect	[95]
P13671	Complement component C6	Opsonization of bacteria/Immunomodulatory effect	[95]
P02748	Complement component C9	Opsonization of bacteria/Immunomodulatory effect	[95]
P00751	Complement factor B	Opsonization of bacteria/Immunomodulatory effect	[95]
P08603	Complement factor H	Opsonization of bacteria/Immunomodulatory effect	[95]
P05156	Complement factor I	Opsonization of bacteria/Immunomodulatory effect	[95]

**Table 3:** Cytokines with increased amount in saliva of patients with OSCC compared with controls. The functions of the proteins in the chemical barrier are indicated as well.

Uniprot Entry	Protein Name	Function in the Chemical Barrier	Reference
P01583	Interleukin-1 alpha	Immunomodulatory effect	[96]
P01584	Interleukin-1 beta	Immunomodulatory effect	[96]
P22301	Interleukin-10	Immunomodulatory effect	[96]
P35225	Interleukin-13	Immunomodulatory effect	[96]
P05231	Interleukin-6	Immunomodulatory effect	[96]
P10145	Interleukin-8	Immunomodulatory effect	[96]
P01375	Tumor necrosis factor	Immunomodulatory effect	[96]

**Table 4:** AMPs with decreased amounts in saliva of patients with OSCC compared with controls. The functions of the proteins in the chemical barrier are indicated as well.

Uniprot Entry	Protein Name	Function in the Chemical Barrier	Reference
P0DUB6	Alpha-amylase 1A	Modulation of biofilm formation	[97]
P0DTE7	Alpha-amylase 1B	Modulation of biofilm formation	[97]
P0DTE8	Alpha-amylase 1C	Modulation of biofilm formation	[97]
P17213	Bactericidal permeability-increasing protein	Antimicrobial activity	[20]
P61769	Beta-2-microglobulin	Immunomodulatory effect/antimicrobial activity	[98,99]
P06865	Beta-hexosaminidase subunit alpha	Antimicrobial activity	[100]
Q96DR5	BPI fold-containing family A member 2	Antimicrobial activity	[101]
Q8N4F0	BPI fold-containing family B member 2	Antimicrobial activity	[102]
Q13231	Chitotriosidase-1	Antifungal activity	[103]
P01040	Cystatin-A	Protease inhibitor	[104]
P04080	Cystatin-B	Protease inhibitor	[104]
P01034	Cystatin-C	Protease inhibitor	[104]
P01036	Cystatin-S	Protease inhibitor	[104]
P09228	Cystatin-SA	Protease inhibitor	[104]
P01037	Cystatin-SN	Protease inhibitor	[104]
Q9NZ08	Endoplasmic reticulum aminopeptidase 1	Protease activity	[105]
Q9GZZ8	Extracellular glycoprotein lacritin	Antimicrobial activity	[106]
Q01469	Fatty acid-binding protein 5	Immunomodulatory effect	[107]
P09211	Glutathione S-transferase P	Immunomodulatory effect	[108]
Q9UBX7	Kallikrein-11	Protease activity	[109]
P42785	Lysosomal Pro-X carboxypeptidase	Protease activity	[110]
P61626	Lysozyme C	Antimicrobial activity	[20]
P59665	Neutrophil defensin 1	Antimicrobial activity	[20]
P26447	Protein S100-A4	Immunomodulatory effect	[87]
Q9NQ38	Serine protease inhibitor Kazal-type 5	Protease inhibitor	[111]
Q4VAX6	Serpin peptidase inhibitor, clade B (Ovalbumin), member 10	Protease inhibitor	[112]
P62328	Thymosin beta-4	Antimicrobial activity	[113]
O60235	Transmembrane protease serine 11D	Protease activity	[114]

**Table 5:** AMPs with contradictory expression profiles in the saliva of patients with OSCC compared with controls. Different studies showed elevated or decreased expression in saliva. The functions of the proteins in the chemical barrier are indicated as well.

Uniprot Entry	Protein Name	Function	Reference
P14174	Macrophage migration inhibitory factor	Antimicrobial activity	[115]
Q9HC84	Mucin-5B	Antimicrobial activity	[77]
P29508	Serpin B3	Protease inhibitor	[82]
P36952	Serpin B5	Protease inhibitor	[82]
P25311	Zinc-alpha-2-glycoprotein	Immunomodulatory effect	[116]

**Table 6:** List of datasets used in this study. The dataset identifier and the name of the source database along with references are listed for each processed dataset.

Dataset Identifier	Source Database	Reference	Dataset Identifier	Source Database	Reference
29632809	PubMed	[27]	21035601	PubMed	[143]
31350970	PubMed	[144]	20138569	PubMed	[145]
29199150	PubMed	[146]	18829504	PubMed	[147]
28545132	PubMed	[26]	PXD020263	ProteomeXchange	[148]
28235782	PubMed	[22]	PXD015722	ProteomeXchange	[25]
26847061	PubMed	[149]	PXD008654	ProteomeXchange	[150]
26552850	PubMed	[23]	PXD012436	ProteomeXchange	[151]
26538482	PubMed	[152]	18617144	PubMed	[153]
26205615	PubMed	[24]	36412636	PubMed	[154]
24863804	PubMed	[155]	34830096	PubMed	[156]
24708169	PubMed	[157]	32899735	PubMed	[158]
23784731	PubMed	[159]	31987131	PubMed	[160]
22301830	PubMed	[161]	31804537	PubMed	[162]
21497587	PubMed	[163]	31109866	PubMed	[164]
21109482	PubMed	[165]	30169911	PubMed	[166]

## Data Availability

Publicly available datasets were analyzed in this study. These data can be found here: http://proteomecentral.proteomexchange.org/cgi/GetDataset (accessed on 8 August 2023), PXD020263, PXD015722, PXD008654, and PXD012436; and https://pubmed.ncbi.nlm.nih.gov/ (accessed on 8 August 2023), 29632809, 31350970, 29199150, 28545132, 28235782, 26847061, 26552850, 26538482, 26205615, 24863804, 24708169, 23784731, 22301830, 21497587, 21109482, 21035601, 20138569, 18829504, 18617144, 36412636, 34830096, 32899735, 31987131, 31804537, 31109866, and 30169911.

## Supplementary Materials

The following supporting information can be downloaded at: https://www.mdpi.com/article/10.3390/ijms241713657/s1, Figure S1: Network view of the interaction network of the upregulated chemical barrier proteins in saliva from patients with OSCC; Figure S2: Network view of the interaction network of the downregulated chemical barrier proteins in saliva from patients with OSCC; Table S1: Results of the ClueGO analysis of the upregulated proteins; Table S2: Results of the ClueGO analysis of the downregulated proteins.

## References

[1] Massano J., Regateiro F.S., Januário G., Ferreira A. (2006). Oral squamous cell carcinoma: Review of prognostic and predictive factors. Oral Surg. Oral Med. Oral Pathol. Oral Radiol. Endodontol..

[2] Argiris A., Karamouzis M.V., Raben D., Ferris R.L. (2008). Head and neck cancer. Lancet.

[3] Ganesh D., Sreenivasan P., Öhman J., Wallström M., Braz-Silva P.H., Giglio D., Kjeller G., Hasséus B. (2018). Potentially Malignant Oral Disorders and Cancer Transformation. Anticancer Res..

[4] Bray F., Ferlay J., Soerjomataram I., Siegel R.L., Torre L.A., Jemal A. (2018). Global cancer statistics 2018: GLOBOCAN estimates of incidence and mortality worldwide for 36 cancers in 185 countries. CA. Cancer J. Clin..

[5] Diz P., Meleti M., Diniz-Freitas M., Vescovi P., Warnakulasuriya S., Johnson N.W., Kerr A.R. (2017). Oral and pharyngeal cancer in Europe: Incidence, mortality and trends as presented to the Global Oral Cancer Forum. Transl. Res. Oral Oncol..

[6] Zhang H., Dziegielewski P.T., Biron V.L., Szudek J., Al-Qahatani K.H., O’Connell D.A., Harris J.R., Seikaly H. (2013). Survival outcomes of patients with advanced oral cavity squamous cell carcinoma treated with multimodal therapy: A multi-institutional analysis. J. Otolaryngol. Head Neck Surg..

[7] Carreras-Torras C., Gay-Escoda C. (2015). Techniques for early diagnosis of oral squamous cell carcinoma: Systematic review. Med. Oral Patol. Oral Cir. Bucal.

[8] Chang J.T., Wang H.-M., Chang K.-W., Chen W.-H., Wen M.-C., Hsu Y.-M., Yung B.Y.-M., Chen I.-H., Liao C.-T., Hsieh L.-L. (2005). Identification of differentially expressed genes in oral squamous cell carcinoma (OSCC): Overexpression of NPM, CDK1 and NDRG1 and underexpression of CHES1. Int. J. Cancer.

[9] Gandini S., Botteri E., Iodice S., Boniol M., Lowenfels A.B., Maisonneuve P., Boyle P. (2008). Tobacco smoking and cancer: A meta-analysis. Int. J. Cancer.

[10] Bagnardi V., Blangiardo M., La Vecchia C., Corrao G. (2001). A meta-analysis of alcohol drinking and cancer risk. Br. J. Cancer.

[11] Rosenquist K. (2005). Risk factors in oral and oropharyngeal squamous cell carcinoma: A population-based case-control study in southern Sweden. Swed. Dent. J. Suppl..

[12] Gupta S., Gupta S. (2015). Role of human papillomavirus in oral squamous cell carcinoma and oral potentially malignant disorders: A review of the literature. Indian J. Dent..

[13] Li Q., Hu Y., Zhou X., Liu S., Han Q., Cheng L. (2020). Role of Oral Bacteria in the Development of Oral Squamous Cell Carcinoma. Cancers.

[14] Robayo D.A.G., Erira H.A.T., Jaimes F.O.G., Torres A.M., Galindo A.I.C. (2019). Oropharyngeal Squamous Cell Carcinoma: Human Papilloma Virus Coinfection with Streptococcus anginosus. Braz. Dent. J..

[15] Humphrey S.P., Williamson R.T. (2001). A review of saliva: Normal composition, flow, and function. J. Prosthet. Dent..

[16] Henskens Y.M., van der Velden U., Veerman E.C., Nieuw Amerongen A.V. (1993). Protein, albumin and cystatin concentrations in saliva of healthy subjects and of patients with gingivitis or periodontitis. J. Periodontal Res..

[17] Kalló G., Kumar A., Tőzsér J., Csősz É. (2022). Chemical Barrier Proteins in Human Body Fluids. Biomedicines.

[18] Kumar P., Kizhakkedathu J.N., Straus S.K. (2018). Antimicrobial Peptides: Diversity, Mechanism of Action and Strategies to Improve the Activity and Biocompatibility In Vivo. Biomolecules.

[19] Epand R.M., Vogel H.J. (1999). Diversity of antimicrobial peptides and their mechanisms of action. Biochim. Biophys. Acta.

[20] Wiesner J., Vilcinskas A. (2010). Antimicrobial peptides: The ancient arm of the human immune system. Virulence.

[21] Csősz É., Kalló G., Márkus B., Deák E., Csutak A., Tőzsér J. (2017). Quantitative body fluid proteomics in medicine—A focus on minimal invasiveness. J. Proteom..

[22] Chen Y.T., Chen H.W., Wu C.F., Chu L.J., Chiang W.F., Wu C.C., Yu J.S., Tsai C.H., Liang K.H., Chang Y.S. (2017). Development of a Multiplexed Liquid Chromatography Multiple-Reaction-Monitoring Mass Spectrometry (LC-MRM/MS) Method for Evaluation of Salivary Proteins as Oral Cancer Biomarkers. Mol. Cell. Proteom..

[23] Kawahara R., Bollinger J.G., Rivera C., Ribeiro A.C.P., Brandão T.B., Leme A.F.P., Maccoss M.J. (2016). A targeted proteomic strategy for the measurement of oral cancer candidate biomarkers in human saliva. Proteomics.

[24] Wu C.C., Chu H.W., Hsu C.W., Chang K.P., Liu H.P. (2015). Saliva proteome profiling reveals potential salivary biomarkers for detection of oral cavity squamous cell carcinoma. Proteomics.

[25] Sivadasan P., Gupta M.K., Sathe G., Sudheendra H.V., Sunny S.P., Renu D., Hari P.S., Gowda H., Suresh A., Kuriakose M.A. (2020). Salivary proteins from dysplastic leukoplakia and oral squamous cell carcinoma and their potential for early detection. J. Proteom..

[26] Csosz E., Lábiscsák P., Kalló G., Márkus B., Emri M., Szabó A., Tar I., Tozsér J., Kiss C., Márton I. (2017). Proteomics investigation of OSCC-specific salivary biomarkers in a Hungarian population highlights the importance of identification of population-tailored biomarkers. PLoS ONE.

[27] Csősz É., Márkus B., Darula Z., Medzihradszky K.F., Nemes J., Szabó E., Tőzsér J., Kiss C., Márton I. (2018). Salivary proteome profiling of oral squamous cell carcinoma in a Hungarian population. FEBS Open Bio..

[28] Márton I.J., Horváth J., Lábiscsák P., Márkus B., Dezső B., Szabó A., Tar I., Piffkó J., Jakus P., Barabás J. (2019). Salivary IL-6 mRNA is a Robust Biomarker in Oral Squamous Cell Carcinoma. J. Clin. Med..

[29] Scholtz B., Vo Minh D., Kiss C., Tar I., Kumar A., Tőzsér J., Csősz É., Márton I. (2020). Examination of Oral Squamous Cell Carcinoma and Precancerous Lesions Using Proximity Extension Assay and Salivary RNA Quantification. Biomedicines.

[30] Vizcaíno J.A., Deutsch E.W., Wang R., Csordas A., Reisinger F., Ríos D., Dianes J.A., Sun Z., Farrah T., Bandeira N. (2014). ProteomeXchange provides globally coordinated proteomics data submission and dissemination. Nat. Biotechnol..

[31] PubMed. www.pubmed.ncbi.nlm.nih.gov.

[32] Kumar A., Doan V.M., Kunkli B., Csősz É. (2021). Construction of Unified Human Antimicrobial and Immunomodulatory Peptide Database and Examination of Antimicrobial and Immunomodulatory Peptides in Alzheimer’s Disease Using Network Analysis of Proteomics Datasets. Front. Genet..

[33] Ceciliani F., Lecchi C. (2019). The Immune Functions of α 1 Acid Glycoprotein. Curr. Protein Pept. Sci..

[34] Dimberg J., Ström K., Löfgren S., Zar N., Hugander A., Matussek A. (2011). Expression of the serine protease inhibitor serpinA3 in human colorectal adenocarcinomas. Oncol. Lett..

[35] Janciauskiene S., Wrenger S., Immenschuh S., Olejnicka B., Greulich T., Welte T., Chorostowska-Wynimko J. (2018). The multifaceted effects of Alpha1-Antitrypsin on neutrophil functions. Front. Pharmacol..

[36] Cederfur C., Salomonsson E., Nilsson J., Halim A., Öberg C.T., Larson G., Nilsson U.J., Leffler H. (2008). Different affinity of galectins for human serum glycoproteins: Galectin-3 binds many protease inhibitors and acute phase proteins. Glycobiology.

[37] Singh S., Saleem S., Reed G.L. (2020). Alpha2-Antiplasmin: The Devil You Don’t Know in Cerebrovascular and Cardiovascular Disease. Front. Cardiovasc. Med..

[38] Wang H., Sama A.E. (2012). Anti-inflammatory role of fetuin-A in injury and infection. Curr. Mol. Med..

[39] Vandooren J., Itoh Y. (2021). Alpha-2-Macroglobulin in Inflammation, Immunity and Infections. Front. Immunol..

[40] Mulligan M.S., Lentsch A.B., Huber-Lang M., Guo R.F., Sarma V., Wright C.D., Ulich T.R., Ward P.A. (2000). Anti-inflammatory effects of mutant forms of secretory leukocyte protease inhibitor. Am. J. Pathol..

[41] Roemisch J., Gray E., Hoffmann J.N., Wiedermann C.J., Kalina U. (2002). Antithrombin: A new look at the actions of a serine protease inhibitor. Blood Coagul. Fibrinolysis.

[42] Tada N., Sakamoto T., Kagami A., Mochizuki K., Kurosaka K. (1993). Antimicrobial activity of lipoprotein particles containing apolipoprotein Al. Mol. Cell. Biochem..

[43] Recalde D., Ostos M.A., Badell E., Garcia-Otin A.L., Pidoux J., Castro G., Zakin M.M., Scott-Algara D. (2004). Human apolipoprotein A-IV reduces secretion of proinflammatory cytokines and atherosclerotic effects of a chronic infection mimicked by lipopolysaccharide. Arterioscler. Thromb. Vasc. Biol..

[44] Gaglione R., Cesaro A., Dell’Olmo E., Della Ventura B., Casillo A., Di Girolamo R., Velotta R., Notomista E., Veldhuizen E.J.A., Corsaro M.M. (2019). Effects of human antimicrobial cryptides identified in apolipoprotein B depend on specific features of bacterial strains. Sci. Rep..

[45] Zewinger S., Reiser J., Jankowski V., Alansary D., Hahm E., Triem S., Klug M., Schunk S.J., Schmit D., Kramann R. (2019). Apolipoprotein C3 induces inflammation and organ damage by alternative inflammasome activation. Nat. Immunol..

[46] Do Carmo S., Jacomy H., Talbot P.J., Rassart E. (2008). Neuroprotective effect of apolipoprotein D against human coronavirus OC43-induced encephalitis in mice. J. Neurosci..

[47] Zhang H., Wu L.M., Wu J. (2011). Cross-talk between apolipoprotein E and cytokines. Mediat. Inflamm..

[48] Fang J., Yao X., Hou M., Duan M., Xing L., Huang J., Wang Y., Zhu B., Chen Q., Wang H. (2020). ApoL1 induces kidney inflammation through RIG-I/NF-κB activation. Biochem. Biophys. Res. Commun..

[49] Serrano M., Morán L., Martinez-Flores J.A., Mancebo E., Pleguezuelo D., Cabrera-Marante O., Delgado J., Serrano A. (2019). Immune Complexes of Beta-2-Glycoprotein I and IgA Antiphospholipid Antibodies Identify Patients With Elevated Risk of Thrombosis and Early Mortality After Heart Transplantation. Front. Immunol..

[50] Liu Y., Bartlett J.A., Di M.E., Bomberger J.M., Chan Y.R., Gakhar L., Mallampalli R.K., McCray P.B., Di Y.P. (2013). SPLUNC1/BPIFA1 Contributes to Pulmonary Host Defense against Klebsiella pneumoniae Respiratory Infection. Am. J. Pathol..

[51] Kono Y. (1995). Apparent antibacterial activity of catalase: Role of lipid hydroperoxide contamination. J. Biochem..

[52] Stafford S.L., Bokil N.J., Achard M.E.S., Kapetanovic R., Schembri M.A., Mcewan A.G., Sweet M.J. (2013). Metal ions in macrophage antimicrobial pathways: Emerging roles for zinc and copper. Biosci. Rep..

[53] Jeong S., Ledee D.R., Gordon G.M., Itakura T., Patel N., Martin A., Fini M.E. (2012). Interaction of clusterin and matrix metalloproteinase-9 and its implication for epithelial homeostasis and inflammation. Am. J. Pathol..

[54] Gardill B.R., Vogl M.R., Lin H.Y., Hammond G.L., Muller Y.A. (2012). Corticosteroid-Binding Globulin: Structure-Function Implications from Species Differences. PLoS ONE.

[55] Sproston N.R., Ashworth J.J. (2018). Role of C-Reactive Protein at Sites of Inflammation and Infection. Front. Immunol..

[56] Torrent M., de la Torre B.G., Nogués V.M., Andreu D., Boix E. (2009). Bactericidal and membrane disruption activities of the eosinophil cationic protein are largely retained in an N-terminal fragment. Biochem. J..

[57] Gong Y., Yu Z., Gao Y., Deng L., Wang M., Chen Y., Li J., Cheng B. (2018). FABP4 inhibitors suppress inflammation and oxidative stress in murine and cell models of acute lung injury. Biochem. Biophys. Res. Commun..

[58] Ko Y.P., Flick M.J. (2016). Fibrinogen Is at the Interface of Host Defense and Pathogen Virulence in Staphylococcus aureus Infection. Semin. Thromb. Hemost..

[59] Loimaranta V., Hepojoki J., Laaksoaho O., Pulliainen A.T. (2018). Galectin-3-binding protein: A multitask glycoprotein with innate immunity functions in viral and bacterial infections. J. Leukoc. Biol..

[60] Ullrich A., Sures I., D’Egidio M., Jallal B., Powell T.J., Herbst R., Dreps A., Azam M., Rubinstein M., Natoli C. (1994). The secreted tumor-associated antigen 90K is a potent immune stimulator. J. Biol. Chem..

[61] Bucki R., Janmey P.A. (2006). Interaction of the gelsolin-derived antibacterial PBP 10 peptide with lipid bilayers and cell membranes. Antimicrob. Agents Chemother..

[62] Hughes M.M., McGettrick A.F., O’Neill L.A.J. (2017). Glutathione and Glutathione Transferase Omega 1 as Key Posttranslational Regulators in Macrophages. Microbiol. Spectr..

[63] Gao X., Wang X., Pham T.H., Feuerbacher L.A., Lubos M.L., Huang M., Olsen R., Mushegian A., Slawson C., Hardwidge P.R. (2013). NleB, a bacterial effector with glycosyltransferase activity, targets GAPDH function to inhibit NF-κB activation. Cell Host Microbe.

[64] MacKellar M., Vigerust D.J. (2016). Role of Haptoglobin in Health and Disease: A Focus on Diabetes. Clin. Diabetes.

[65] Drain J., Bishop J.R., Hajduk S.L. (2001). Haptoglobin-related protein mediates trypanosome lytic factor binding to trypanosomes. J. Biol. Chem..

[66] Parish C.A., Jiang H., Tokiwa Y., Berova N., Nakanishi K., McCabe D., Zuckerman W., Xia M.M., Gabay J.E. (2001). Broad-spectrum antimicrobial activity of hemoglobin. Bioorg. Med. Chem..

[67] Yin X., Li X., Chen N., Mu L., Wu H., Yang Y., Han K., Huang Y., Wang B., Jian J. (2021). Hemopexin as an acute phase protein regulates the inflammatory response against bacterial infection of Nile tilapia (*Oreochromis niloticus*). Int. J. Biol. Macromol..

[68] He L., Vicente C.P., Westrick R.J., Eitzman D.T., Tollefsen D.M. (2002). Heparin cofactor II inhibits arterial thrombosis after endothelial injury. J. Clin. Investig..

[69] Rydengård V., Olsson A.K., Mörgelin M., Schmidtchen A. (2007). Histidine-rich glycoprotein exerts antibacterial activity. FEBS J..

[70] Hoeksema M., Van Eijk M., Haagsman H.P., Hartshorn K.L. (2016). Histones as mediators of host defense, inflammation and thrombosis. Future Microbiol..

[71] Zhuo L., Kimata K. (2008). Structure and function of inter-alpha-trypsin inhibitor heavy chains. Connect. Tissue Res..

[72] Rapala-Kozik M., Karkowska J., Jacher A., Golda A., Barbasz A., Guevara-Lora I., Kozik A. (2008). Kininogen adsorption to the cell surface of *Candida* spp.. Int. Immunopharmacol..

[73] Ben Nasr A., Herwald H., Muller-Esterl W., Bjorck L. (1995). Human kininogens interact with M protein, a bacterial surface protein and virulence determinant. Biochem. J..

[74] Ra H.J., Parks W.C. (2007). Control of Matrix Metalloproteinase Catalytic Activity. Matrix Biol..

[75] Hong J.-S., Greenlee K.J., Pitchumani R., Lee S.-H., Song L., Shan M., Chang S.H., Park P.W., Dong C., Werb Z. (2011). Dual protective mechanisms of matrix metalloproteinases 2 and 9 in immune defense against *Streptococcus pneumoniae*. J. Immunol..

[76] Serrador J.M., Alonso-Lebrero J.L., Del Pozo M.A., Furthmayr H., Schwartz-Albiez R., Calvo J., Lozano F., Sánchez-Madrid F. (1997). Moesin interacts with the cytoplasmic region of intercellular adhesion molecule-3 and is redistributed to the uropod of T lymphocytes during cell polarization. J. Cell Biol..

[77] Linden S.K., Sutton P., Karlsson N.G., Korolik V., McGuckin M.A. (2008). Mucins in the mucosal barrier to infection. Mucosal Immunol..

[78] Brockmann H., Schwarting A., Kriegsmann J., Petrow P., Gaumann A., Müller K.M., Galle P.R., Mayet W. (2002). Proteinase-3 as the major autoantigen of c-ANCA is strongly expressed in lung tissue of patients with Wegener’s granulomatosis. Arthritis Res..

[79] Flo T.H., Smith K.D., Sato S., Rodriguez D.J., Holmes M.A., Strong R.K., Akira S., Aderem A. (2004). Lipocalin 2 mediates an innate immune response to bacterial infection by sequestrating iron. Nature.

[80] Kang D., Liu G., Lundström A., Gelius E., Steiner H. (1998). A peptidoglycan recognition protein in innate immunity conserved from insects to humans. Proc. Natl. Acad. Sci. USA.

[81] Steele F.R., Chader G.J., Johnson L.V., Tombran-Tink J. (1993). Pigment epithelium-derived factor: Neurotrophic activity and identification as a member of the serine protease inhibitor gene family. Proc. Natl. Acad. Sci. USA.

[82] Law R.H.P., Zhang Q., McGowan S., Buckle A.M., Silverman G.A., Wong W., Rosado C.J., Langendorf C.G., Pike R.N., Bird P.I. (2006). An overview of the serpin superfamily. Genome Biol..

[83] Lu X., Kugadas A., Smith-Page K., Lamb J., Lin T., Ru Y., Morley S.C., Fichorova R., Mittal S.K., Chauhan S.K. (2020). Neutrophil L-Plastin Controls Ocular Paucibacteriality and Susceptibility to Keratitis. Front. Immunol..

[84] Tamir A., Gangadharan A., Balwani S., Tanaka T., Patel U., Hassan A., Benke S., Agas A., D’Agostino J., Shin D. (2016). The serine protease prostasin (PRSS8) is a potential biomarker for early detection of ovarian cancer. J. Ovarian Res..

[85] Vetr H., Gebhard W. (1990). Structure of the human alpha 1-microglobulin-bikunin gene. Biol. Chem. Hoppe. Seyler..

[86] Zhang L., Zhu T., Miao H., Liang B. (2021). The Calcium Binding Protein S100A11 and Its Roles in Diseases. Front. cell Dev. Biol..

[87] Xia C., Braunstein Z., Toomey A.C., Zhong J., Rao X. (2018). S100 proteins as an important regulator of macrophage inflammation. Front. Immunol..

[88] Donato R. (2003). Intracellular and extracellular roles of S100 proteins. Microsc. Res. Tech..

[89] Bruhn K.W., Spellberg B. (2015). Transferrin-Mediated Iron Sequestration as a Novel Therapy for Bacterial and Fungal Infections. Curr. Opin. Microbiol..

[90] Gatt M.E., Urieli-Shoval S., Preciado-Patt L., Fridkin M., Calco S., Azar Y., Matzner Y. (1998). Effect of serum amyloid A on selected in vitro functions of isolated human neutrophils. J. Lab. Clin. Med..

[91] Gomis-Rüth F.X., Maskos K., Betz M., Bergner A., Huber R., Suzuki K., Yoshida N., Nagase H., Brew K., Bourenkov G.P. (1997). Mechanism of inhibition of the human matrix metalloproteinase stromelysin-1 by TIMP-1. Nature.

[92] Jirasakuldech B., Schussler G.C., Yap M.G., Drew H., Josephson A., Michl J. (2000). A characteristic serpin cleavage product of thyroxine-binding globulin appears in sepsis sera. J. Clin. Endocrinol. Metab..

[93] Kim H.R., Park J.S., Karabulut H., Yasmin F., Jun C.D. (2021). Transgelin-2: A Double-Edged Sword in Immunity and Cancer Metastasis. Front. Cell Dev. Biol..

[94] Kew R.R. (2019). The Vitamin D binding protein and inflammatory injury: A mediator or sentinel of tissue damage?. Front. Endocrinol..

[95] Merle N.S., Church S.E., Fremeaux-Bacchi V., Roumenina L.T. (2015). Complement system part I-molecular mechanisms of activation and regulation. Front. Immunol..

[96] Zhang J.M., An J. (2007). Cytokines, Inflammation and Pain. Int. Anesthesiol. Clin..

[97] Lahiri D., Nag M., Banerjee R., Mukherjee D., Garai S., Sarkar T., Dey A., Sheikh H.I., Pathak S.K., Edinur H.A. (2021). Amylases: Biofilm Inducer or Biofilm Inhibitor?. Front. Cell. Infect. Microbiol..

[98] Chiou S.J., Ko H.J., Hwang C.C., Hong Y.R. (2021). The Double-Edged Sword of Beta2-Microglobulin in Antibacterial Properties and Amyloid Fibril-Mediated Cytotoxicity. Int. J. Mol. Sci..

[99] Xie J., Yi Q., Uchanska-Ziegler B., Ziegler A. (2003). β2-microglobulin as a potential initiator of inflammatory responses. Trends Immunol..

[100] Koo I.C., Ohol Y.M., Wu P., Morisaki J.H., Cox J.S., Brown E.J. (2008). Role for lysosomal enzyme β-hexosaminidase in the control of mycobacteria infection. Proc. Natl. Acad. Sci. USA.

[101] Prokopovic V., Popovic M., Andjelkovic U., Marsavelski A., Raskovic B., Gavrovic-Jankulovic M., Polovic N. (2014). Isolation, biochemical characterization and anti-bacterial activity of BPIFA2 protein. Arch. Oral Biol..

[102] Huang Y., Wang M., Hong Y., Bu X., Luan G., Wang Y., Li Y., Lou H., Wang C., Zhang L. (2021). Reduced Expression of Antimicrobial Protein Secretory Leukoprotease Inhibitor and Clusterin in Chronic Rhinosinusitis with Nasal Polyps. J. Immunol. Res..

[103] Hall A.J., Quinnell R.J., Raiko A., Lagog M., Siba P., Morroll S., Falcone F.H. (2007). Chitotriosidase deficiency is not associated with human hookworm infection in a Papua New Guinean population. Infect. Genet. Evol..

[104] Zavasnik-Bergant T. (2008). Cystatin protease inhibitors and immune functions. Front. Biosci..

[105] Haroon N., Inman R.D. (2010). Endoplasmic reticulum aminopeptidases: Biology and pathogenic potential. Nat. Rev. Rheumatol..

[106] McKown R.L., Coleman Frazier E.V., Zadrozny K.K., Deleault A.M., Raab R.W., Ryan D.S., Sia R.K., Lee J.K., Laurie G.W. (2014). A cleavage-potentiated fragment of tear lacritin is bactericidal. J. Biol. Chem..

[107] Suojalehto H., Kinaret P., Kilpeläinen M., Toskala E., Ahonen N., Wolff H., Alenius H., Puustinen A. (2015). Level of Fatty Acid Binding Protein 5 (FABP5) Is Increased in Sputum of Allergic Asthmatics and Links to Airway Remodeling and Inflammation. PLoS ONE.

[108] Wu Y., Fan Y., Xue B., Luo L., Shen J., Zhang S., Jiang Y., Yin Z. (2006). Human glutathione S-transferase P1-1 interacts with TRAF2 and regulates TRAF2–ASK1 signals. Oncogene.

[109] Diamandis E.P., Okui A., Mitsui S., Luo L.-Y., Soosaipillai A., Grass L., Nakamura T., Howarth D.J.C., Yamaguchi N. (2002). Human Kallikrein 11: A New Biomarker of Prostate and Ovarian Carcinoma. Cancer Res..

[110] Jeong J.K., Diano S. (2013). Prolyl carboxypeptidase and its inhibitors in metabolism. Trends Endocrinol. Metab..

[111] Chavanas S., Bodemer C., Rochat A., Hamel-Teillac D., Ali M., Irvine A.D., Bonafé J.L., Wilkinson J., Taïeb A., Barrandon Y. (2000). Mutations in SPINK5, encoding a serine protease inhibitor, cause Netherton syndrome. Nat. Genet..

[112] Turato C., Pontisso P. (2015). SERPINB3 (serpin peptidase inhibitor, clade B (ovalbumin), member 3). Atlas Genet. Cytogenet. Oncol. Haematol..

[113] Carion T.W., Ebrahim A.S., Alluri S., Ebrahim T., Parker T., Burns J., Sosne G., Berger E.A. (2020). Antimicrobial Effects of Thymosin Beta-4 and Ciprofloxacin Adjunctive Therapy in Pseudomonas aeruginosa Induced Keratitis. Int. J. Mol. Sci..

[114] Yasuoka S., Ohnishi T., Kawano S., Tsuchihashi S., Ogawara M., Masuda K.I., Yamaoka K., Takahashi M., Sano T. (1997). Purification, characterization, and localization of a novel trypsin-like protease found in the human airway. Am. J. Respir. Cell Mol. Biol..

[115] Oddo M., Calandra T., Bucala R., Meylan P.R.A. (2005). Macrophage migration inhibitory factor reduces the growth of virulent Mycobacterium tuberculosis in human macrophages. Infect. Immun..

[116] Hassan M.I., Waheed A., Yadav S., Singh T.P., Ahmad F. (2009). Prolactin inducible protein in cancer, fertility and immunoregulation: Structure, function and its clinical implications. Cell. Mol. Life Sci..

[117] Szklarczyk D., Gable A.L., Nastou K.C., Lyon D., Kirsch R., Pyysalo S., Doncheva N.T., Legeay M., Fang T., Bork P. (2021). The STRING database in 2021: Customizable protein-protein networks, and functional characterization of user-uploaded gene/measurement sets. Nucleic Acids Res..

[118] Shannon P., Markiel A., Ozier O., Baliga N.S., Wang J.T., Ramage D., Amin N., Schwikowski B., Ideker T. (2003). Cytoscape: A software environment for integrated models of biomolecular interaction networks. Genome Res..

[119] Bindea G., Mlecnik B., Hackl H., Charoentong P., Tosolini M., Kirilovsky A., Fridman W.-H., Pagès F., Trajanoski Z., Galon J. (2009). ClueGO: A Cytoscape plug-in to decipher functionally grouped gene ontology and pathway annotation networks. Bioinformatics.

[120] Bindea G., Galon J., Mlecnik B. (2013). CluePedia Cytoscape plugin: Pathway insights using integrated experimental and in silico data. Bioinformatics.

[121] Chin C.-H., Chen S.-H., Wu H.-H., Ho C.-W., Ko M.-T., Lin C.-Y. (2014). cytoHubba: Identifying hub objects and sub-networks from complex interactome. BMC Syst. Biol..

[122] Theofilou D.V.I., Ghita D.I., Elnaggar D.M., Chaisuparat D.R., Dyalram D.D., Ord P.R.A., Lubek D.J.E., Younis D.R.H. (2022). Stromal inflammatory subtypes of oral squamous cell carcinoma correlate with patient clinical characteristics, demographics and gene expression. Oral Surg. Oral Med. Oral Pathol. Oral Radiol..

[123] Goertzen C., Mahdi H., Laliberte C., Meirson T., Eymael D., Gil-Henn H., Magalhaes M. (2018). Oral inflammation promotes oral squamous cell carcinoma invasion. Oncotarget.

[124] Wu M., Ye P., Zhang W., Zhu H., Yu H. (2022). Prognostic role of an inflammation scoring system in radical resection of oral squamous cell carcinoma. BMC Oral Health.

[125] Navale A.M., Deshpande A., Mistry B., Chauhan P., Bhagat C. (2023). Salivary protein biomarkers for diagnosis of oral squamous cell carcinoma. Curr. Cancer Drug Targets.

[126] Feng Y., Li Q., Chen J., Yi P., Xu X., Fan Y., Cui B., Yu Y., Li X., Du Y. (2019). Salivary protease spectrum biomarkers of oral cancer. Int. J. Oral Sci..

[127] Agbowuro A.A., Huston W.M., Gamble A.B., Tyndall J.D.A. (2018). Proteases and protease inhibitors in infectious diseases. Med. Res. Rev..

[128] Piyarathne N.S., Weerasekera M.M., Fonseka P.F.D., Karunatilleke A.H.T.S., Liyanage R.L.P.R., Jayasinghe R.D., De Silva K., Yasawardene S., Gupta E., Jayasinghe J.A.P. (2023). Salivary Interleukin Levels in Oral Squamous Cell Carcinoma and Oral Epithelial Dysplasia: Findings from a Sri Lankan Study. Cancers.

[129] Zackular J.P., Chazin W.J., Skaar E.P. (2015). Nutritional Immunity: S100 Proteins at the Host-Pathogen Interface. J. Biol. Chem..

[130] Ajona D., Pajares M.J., Chiara M.D., Rodrigo J.P., Jantus-Lewintre E., Camps C., Suarez C., Bagán J.V., Montuenga L.M., Pio R. (2015). Complement activation product C4d in oral and oropharyngeal squamous cell carcinoma. Oral Dis..

[131] Ain D., Shaikh T., Manimala S., Ghebrehiwet B. (2021). The role of complement in the tumor microenvironment. Fac. Rev..

[132] Dominiczak M.H., Caslake M.J. (2011). Apolipoproteins: Metabolic role and clinical biochemistry applications. Ann. Clin. Biochem..

[133] Dell’Olmo E., Gaglione R., Sabbah M., Schibeci M., Cesaro A., Di Girolamo R., Porta R., Arciello A. (2021). Host defense peptides identified in human apolipoprotein B as novel food biopreservatives and active coating components. Food Microbiol..

[134] Li X., Liu Y., Yang X., Li C., Song Z. (2022). The Oral Microbiota: Community Composition, Influencing Factors, Pathogenesis, and Interventions. Front. Microbiol..

[135] Dewhirst F.E., Chen T., Izard J., Paster B.J., Tanner A.C.R., Yu W.H., Lakshmanan A., Wade W.G. (2010). The human oral microbiome. J. Bacteriol..

[136] Castagnola M., Scarano E., Passali G.C., Messana I., Cabras T., Iavarone F., Cintio G.D., Fiorita A., Corso E.D., Paludetti G. (2017). Salivary biomarkers and proteomics:future diagnostic and clinical utilities. Acta Otorhinolaryngol. Ital..

[137] Jiang X., Zhang Y., Wang H., Wang Z., Hu S., Cao C., Xiao H. (2022). In-Depth Metaproteomics Analysis of Oral Microbiome for Lung Cancer. Research.

[138] Grassl N., Kulak N.A., Pichler G., Geyer P.E., Jung J., Schubert S., Sinitcyn P., Cox J., Mann M. (2016). Ultra-deep and quantitative saliva proteome reveals dynamics of the oral microbiome. Genome Med..

[139] Rabe A., Gesell Salazar M., Michalik S., Fuchs S., Welk A., Kocher T., Völker U. (2019). Metaproteomics analysis of microbial diversity of human saliva and tongue dorsum in young healthy individuals. J. Oral Microbiol..

[140] Bostanci N., Grant M., Bao K., Silbereisen A., Hetrodt F., Manoil D., Belibasakis G.N. (2021). Metaproteome and metabolome of oral microbial communities. Periodontol.

[141] Garley M., Dziemiańczyk-Pakieła D., Ratajczak-Wrona W., Pryczynicz A., Nowak K., Łazarczyk B., Jabłońska E. (2022). NETs biomarkers in saliva and serum OSCC patients: One hypothesis, two conclusions. Adv. Med. Sci..

[142] Nijakowski K., Gruszczyński D., Kopała D., Surdacka A. (2022). Salivary Metabolomics for Oral Squamous Cell Carcinoma Diagnosis: A Systematic Review. Metabolites.

[143] Jou Y.J., Lin C.D., Lai C.H., Chen C.H., Kao J.Y., Chen S.Y., Tsai M.H., Huang S.H., Lin C.W. (2010). Proteomic identification of salivary transferrin as a biomarker for early detection of oral cancer. Anal. Chim. Acta.

[144] G D., Nandan S.R.K., Kulkarni P.G. (2019). Salivary Tumour Necrosis Factor-α as a Biomarker in Oral Leukoplakia and Oral Squamous Cell Carcinoma. Asian Pac. J. Cancer Prev..

[145] Wu J.Y., Yi C., Chung H.R., Wang D.J., Chang W.C., Lee S.Y., Lin C.T., Yang Y.C., Yang W.C.V. (2010). Potential biomarkers in saliva for oral squamous cell carcinoma. Oral Oncol..

[146] Heawchaiyaphum C., Pientong C., Phusingha P., Vatanasapt P., Promthet S., Daduang J., Teeramatwanich W., Kongyingyoes B., Chuerduangphui J., Ekalaksananan T. (2018). Peroxiredoxin-2 and zinc-alpha-2-glycoprotein as potentially combined novel salivary biomarkers for early detection of oral squamous cell carcinoma using proteomic approaches. J. Proteom..

[147] Hu S., Arellano M., Boontheung P., Wang J., Zhou H., Jiang J., Elashoff D., Wei R., Loo J.A., Wong D.T. (2008). Salivary proteomics for oral cancer biomarker discovery. Clin. Cancer Res..

[148] Jain A., Kotimoole C.N., Ghoshal S., Bakshi J., Chatterjee A., Prasad T.S.K., Pal A. (2021). Identification of potential salivary biomarker panels for oral squamous cell carcinoma. Sci. Rep..

[149] Gleber-Netto F.O., Yakob M., Li F., Feng Z., Dai J., Kao H.K., Chang Y.L., Chang K.P., Wong D.T.W. (2016). Salivary Biomarkers for Detection of Oral Squamous Cell Carcinoma in a Taiwanese Population. Clin. Cancer Res..

[150] Hsu C.W., Chang K.P., Huang Y., Liu H.P., Hsueh P.C., Gu P.W., Yen W.C., Wu C.C. (2019). Proteomic Profiling of Paired Interstitial Fluids Reveals Dysregulated Pathways and Salivary NID1 as a Biomarker of Oral Cavity Squamous Cell Carcinoma. Mol. Cell. Proteom..

[151] Lin Y.H., Eguez R.V., Torralba M.G., Singh H., Golusinski P., Golusinski W., Masternak M., Nelson K.E., Freire M., Yu Y. (2019). Self-Assembled STrap for Global Proteomics and Salivary Biomarker Discovery. J. Proteome Res..

[152] Winck F.V., Ribeiro A.C.P., Domingues R.R., Ling L.Y., Riaño-Pachón D.M., Rivera C., Brandão T.B., Gouvea A.F., Santos-Silva A.R., Coletta R.D. (2015). Insights into immune responses in oral cancer through proteomic analysis of saliva and salivary extracellular vesicles. Sci. Rep..

[153] Dowling P., Wormald R., Meleady P., Henry M., Curran A., Clynes M. (2008). Analysis of the saliva proteome from patients with head and neck squamous cell carcinoma reveals differences in abundance levels of proteins associated with tumour progression and metastasis. J. Proteom..

[154] Riccardi G., Bellizzi M.G., Fatuzzo I., Zoccali F., Cavalcanti L., Greco A., Vincentiis M.d., Ralli M., Fiore M., Petrella C. (2022). Salivary Biomarkers in Oral Squamous Cell Carcinoma: A Proteomic Overview. Proteomes.

[155] Jou Y.J., Hua C.H., Lin C.D., Lai C.H., Huang S.H., Tsai M.H., Kao J.Y., Lin C.W. (2014). S100A8 as potential salivary biomarker of oral squamous cell carcinoma using nanoLC-MS/MS. Clin. Chim. Acta.

[156] Manzano-Moreno F.J., Costela-Ruiz V.J., García-Recio E., Olmedo-Gaya M.V., Ruiz C., Reyes-Botella C. (2021). Role of Salivary MicroRNA and Cytokines in the Diagnosis and Prognosis of Oral Squamous Cell Carcinoma. Int. J. Mol. Sci..

[157] Hsu C.W., Yu J.S., Peng P.H., Liu S.C., Chang Y.S., Chang K.P., Wu C.C. (2014). Secretome profiling of primary cells reveals that THBS2 is a salivary biomarker of oral cavity squamous cell carcinoma. J. Proteome Res..

[158] Roi A., Roi C.I., Negruțiu M.L., Riviș M., Sinescu C., Rusu L.C. (2020). The Challenges of OSCC Diagnosis: Salivary Cytokines as Potential Biomarkers. J. Clin. Med..

[159] Jessie K., Jayapalan J.J., Ong K.-C., Abdul Rahim Z.H., Zain R.M., Wong K.-T., Hashim O.H. (2013). Aberrant proteins in the saliva of patients with oral squamous cell carcinoma. Electrophoresis.

[160] Hsiao Y.C., Lin S.Y., Chien K.Y., Chen S.F., Wu C.C., Chang Y.T., Chi L.M., Chu L.J., Chiang W.F., Chien C.Y. (2020). An immuno-MALDI mass spectrometry assay for the oral cancer biomarker, matrix metalloproteinase-1, in dried saliva spot samples. Anal. Chim. Acta.

[161] Elashoff D., Zhou H., Reiss J., Wang J., Xiao H., Henson B., Hu S., Arellano M., Sinha U., Le A. (2012). Prevalidation of salivary biomarkers for oral cancer detection. Cancer Epidemiol. Biomark. Prev..

[162] Faria P.C.B., Carneiro A.P., Binato R., Nascimento R., Santos P.S., Fagundes D., da Silva S.J., Loyola A.M., Abdelhay E., Goulart L.R. (2019). Upregulation of tropomyosin alpha-4 chain in patients’ saliva with oral squamous cell carcinoma as demonstrated by Phage display. Sci. Rep..

[163] Jou Y.J., Lin C.D., Lai C.H., Tang C.H., Huang S.H., Tsai M.H., Chen S.Y., Kao J.Y., Lin C.W. (2011). Salivary zinc finger protein 510 peptide as a novel biomarker for detection of oral squamous cell carcinoma in early stages. Clin. Chim. Acta.

[164] Chattopadhyay I., Panda M. (2019). Recent trends of saliva omics biomarkers for the diagnosis and treatment of oral cancer. J. Oral Biosci..

[165] Brinkmann O., Kastratovic D.A., Dimitrijevic M.V., Konstantinovic V.S., Jelovac D.B., Antic J., Nesic V.S., Markovic S.Z., Martinovic Z.R., Akin D. (2011). Oral squamous cell carcinoma detection by salivary biomarkers in a Serbian population. Oral Oncol..

[166] Shan J., Sun Z., Yang J., Xu J., Shi W., Wu Y., Fan Y., Li H. (2019). Discovery and preclinical validation of proteomic biomarkers in saliva for early detection of oral squamous cell carcinomas. Oral Dis..

