# Supplementary figures and images for: Salivary Chemical Barrier Proteins in Oral Squamous Cell Carcinoma—Alterations in the Defense Mechanism of the Oral Cavity
Source: Int J Mol Sci. 2023 Sep 4;24(17):13657. doi: 10.3390/ijms241713657 (PMC10487546; doi:10.3390/ijms241713657)

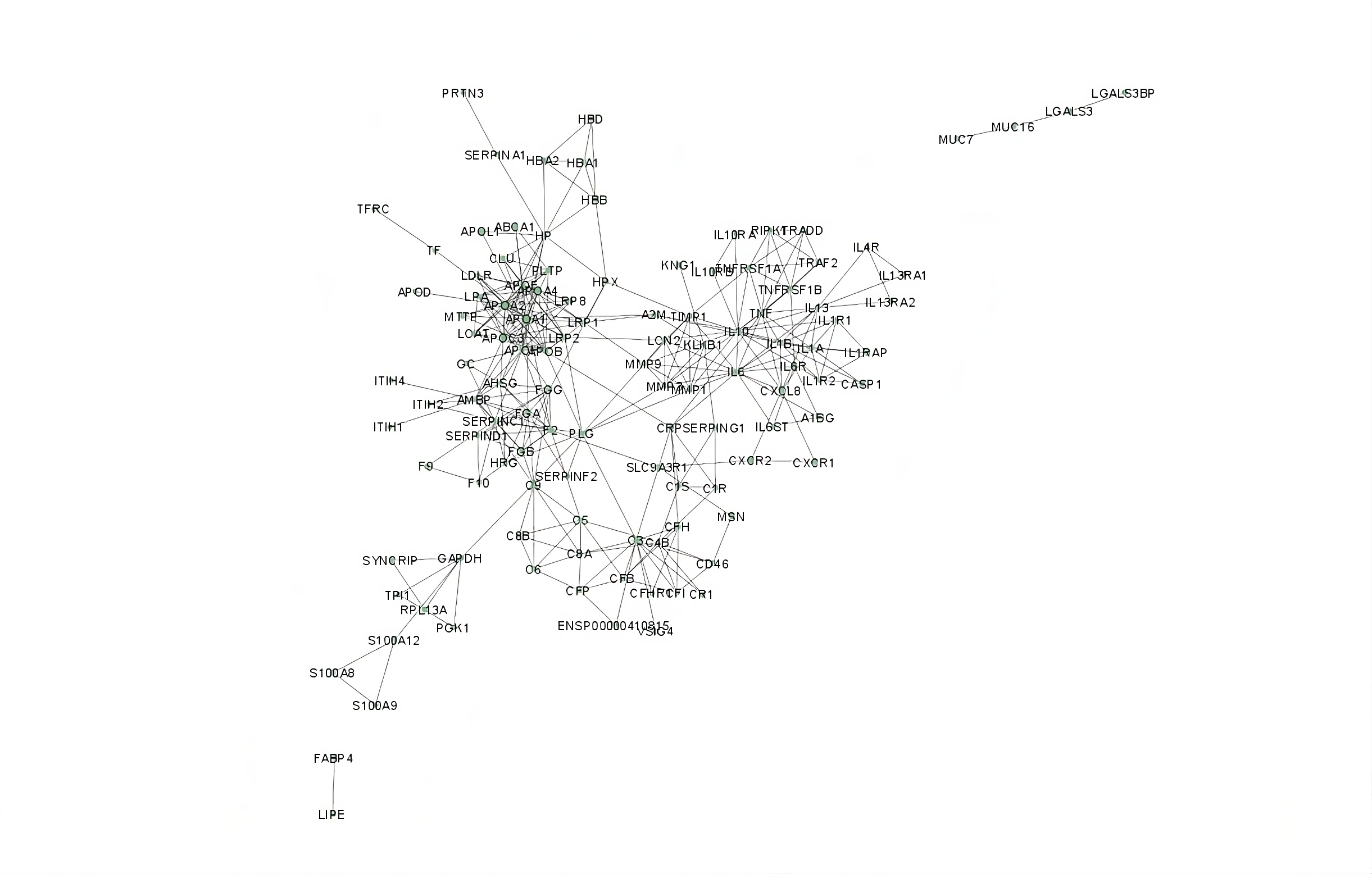

Supplement: Supplementary file 1 [file ijms-24-13657-s001.zip › Figure S1.png]

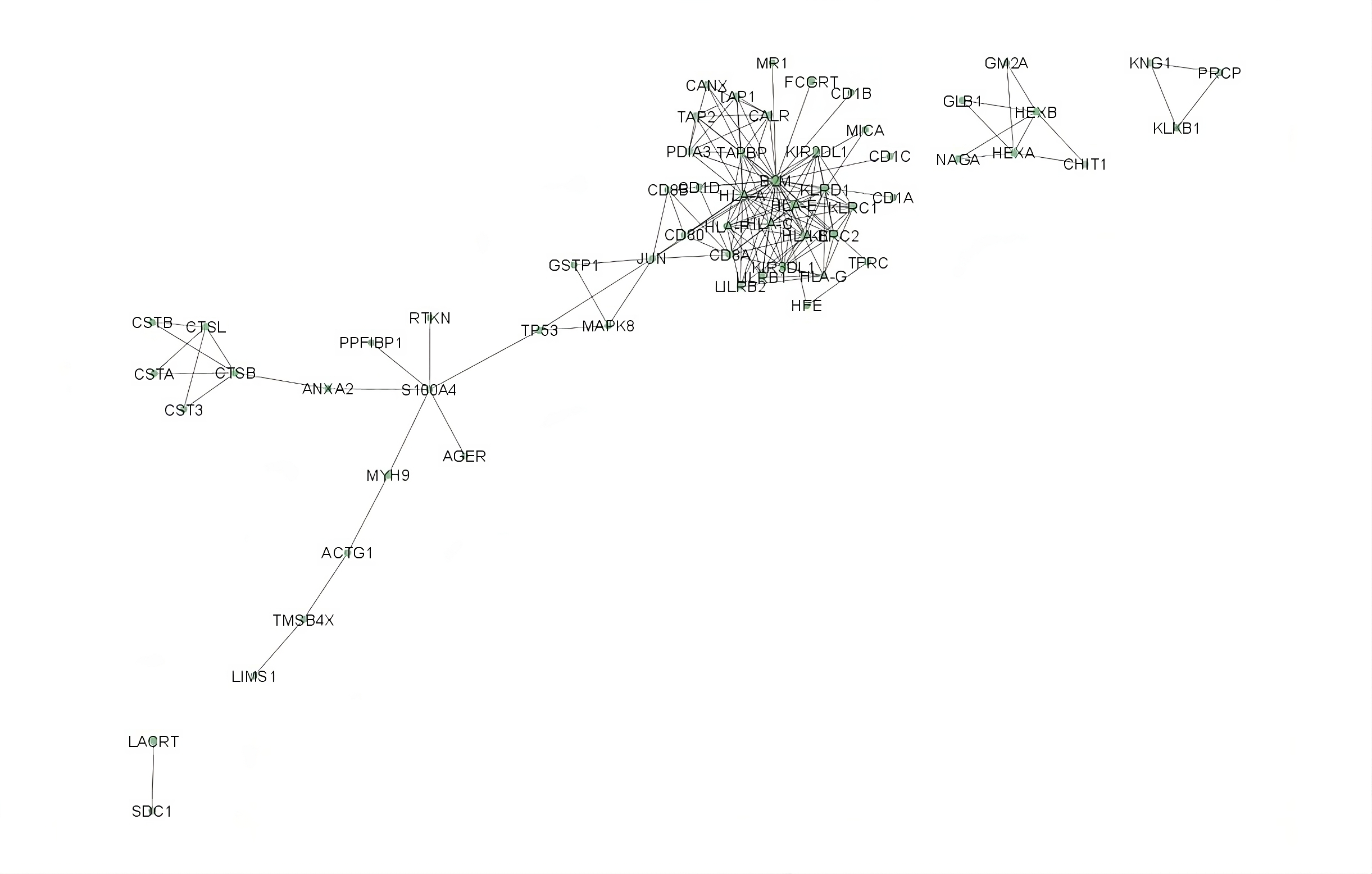

Supplement: Supplementary file 1 [file ijms-24-13657-s001.zip › Figure S2.png]
